# Reconstruction of persistent oronasal communication using an anteriorly based tongue flap following failed cleft palate repair—Report of two cases

**DOI:** 10.1002/ccr3.7066

**Published:** 2023-03-08

**Authors:** Symon Guthua, Krishan Sarna, Martin Kamau, Peter M. Ng'ang'a

**Affiliations:** ^1^ Unit of Oral and Maxillofacial Surgery, Oral Pathology and Oral Medicine, Department of Dental Sciences University of Nairobi Nairobi Kenya; ^2^ Department of Human Anatomy University of Nairobi Nairobi Kenya; ^3^ Unit of Pediatric Dentistry and Orthodontics, Department of Dental Sciences University of Nairobi Nairobi Kenya

**Keywords:** dorsal tongue flap, failed cleft palate repair, persistent cleft palate, persistent oronasal communication, tongue flaps

## Abstract

The tongue flap is a suitable alternative to local mucoperiosteal flaps in closure of wide, persistent oronasal communications, surrounded by scarred and fibrotic tissue as a result of previously attempted palatoplasty. Herein, we report two cases with large recurrent oronasal communication closed using the anteriorly based dorsal tongue flap.

## Graphical abstract

Closure of oronasal communications using mucoperiosteal flaps alone may be impossible and may lead to repeated failure. Due to its tremendous design versatility and high vascularity, the tongue flap can successfully achieve closure of such defects.

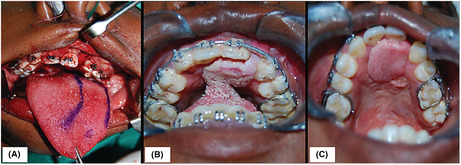

## 1 INTRODUCTION

Cleft palate is one of the most common congenital anomalies of the head and neck region, and has an incidence of 1.7 per 1000 live births in East Africa and affects females slightly more than males.^1^ While small clefts may be asymptomatic, larger ones produce various symptoms such as reflux of food and fluid through the nasal cavity, abnormal speech, deafness due to recurrent middle ear infections, and alterations in craniofacial development. Thus, repair of such defects is essential.^2^ Ideally, treatment begins in the first year after birth and may last up to two decades, involving multiple surgeries, consultation with numerous specialists, and constant follow‐ups. It is needless to say, such intensive therapy has a massive psychological and financial impact on the lives of such patients and their families.^3^ Therefore, the most devastating complication following cleft palate repair is a recurrence of the previously closed opening. This opening may be arbitrarily referred to as a fistula; however, considering the definition of a fistula as an epithelial lined tract connecting two body cavities or one body cavity to the exterior, it does not accurately describe this type of defect. Considering there is no fistulous tract due to the fusion of the nasal mucosa to the oral mucosa, these types of defects are termed oronasal communications (ONCs).^4^

The ONCs are a well‐known postoperative complication of primary cleft palate repair which can occur even in the best of hands and in the most advanced medical settings, thus presenting one of the most difficult challenges that can face the surgeon. They occur at any point along the line of the repaired cleft, and can be classified based on either their location (anterior, midpalate, or at the junction between the hard and soft palate) or their size (small [< 3 mm], medium [3‐5 mm], and large [> 5 mm]).^5^ Factors that play an important role in their development include the cleft type and its size, the underlying medical history of the patient, the operative technique employed, and the surgeon's experience. Recurrent ONCs are extremely challenging to manage successfully, which may be attributed to the palatal mucoperiosteum being scarred as a result of previous surgeries leading to flap necrosis and wound dehiscence which is extremely frustrating to the patient.^6^ Accordingly, an alternative technique is necessary to achieve the successful closure of the defect.

In failures of cleft palate repair resulting in recurrent ONCs, tongue flaps are an ideal alternative to local mucoperiosteal flaps due to their numerous advantages over other types of flaps. Two stages of the procedure can be identified: elevation and suturing of the flap to the margins of the defect, followed 3 weeks later by division of the pedicle.^7^ Morbidity at the donor site is negligible, with both sensation and function remaining intact postoperatively. These merits of the tongue flap make it effective in the closure of ONC which are not amenable to the advancement and rotation of the local mucoperiosteal flaps. In this paper, we describe the cases of two patients that were referred from other centers for specialized evaluation and management following numerous failed surgeries attempting to repair the palatal defects secondary to cleft palate using local mucoperiosteal flaps. Despite previous surgeries, the size of the defects was still quite massive (3.0 and 4.0 cm, respectively) and was surrounded by thick, fibrotic, and scarred tissue. After careful consideration, closure of these defects using the local mucoperiosteal flaps was ruled out, and an alternative method was employed: The anteriorly based dorsal tongue flap which yielded a successful outcome with no further complications.

## 2 CASE PRESENTATIONS

### 2.1 CASE 1

**FIGURE 1:**
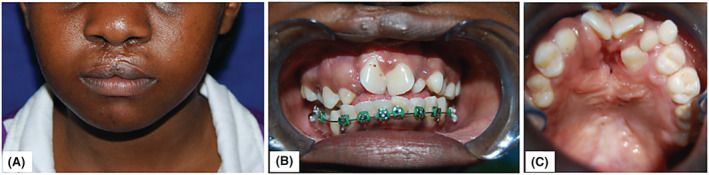
(A) Bilateral scarring and notching of the upper lip after cheiloplasty. (B) Anterior view showing the state of occlusion at the beginning of treatment. Note the presence of alveolar clefts bilaterally. (C) Palatal view revealing anterior crowding accompanied by an anteriorly located oronasal communication surrounded by fibrotic scar tissue resulting from previous surgical attempts.

**FIGURE 2:**
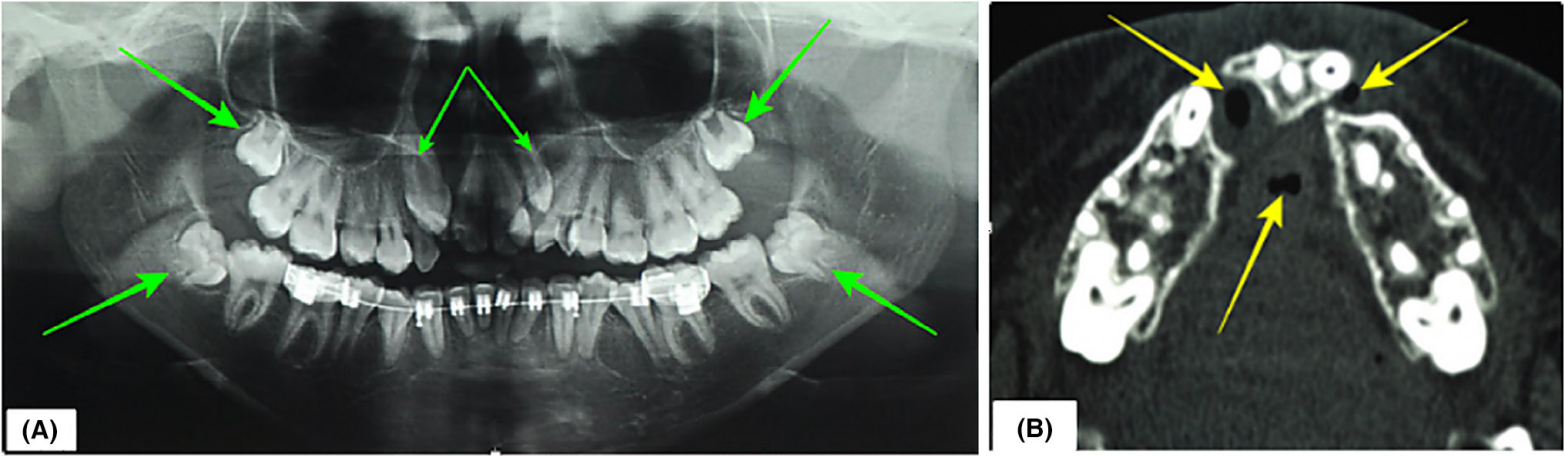
(A) Panoramic radiograph showing the anterior maxillary crowding and multiple impacted teeth (orange arrows). (B) Axial section computed tomography scan showing discontinuity of the alveolus with fistulae (yellow arrows).

A 16‐year‐old African female was referred for management of persistent ONC secondary to bilateral cleft lip and palate. The chief complaint was difficulty in feeding due to oronasal regurgitation, and misalignment of the anterior teeth resulting in a reduction in her social presence and self‐confidence. The patient had previously undergone cheiloplasty for the cleft lip and a total of three attempted unsuccessful surgeries to repair the palatal clefts. Extraoral examination revealed an unsightly notching and scarring of the upper lip. Intraoral examination revealed a host of findings: Class III skeletal relationship, anterior crossbite, bilateral posterior crossbite, and accompanying palatonasal and labionasal fistulae. Furthermore, 12, 13, 18, 22, 25, 28, 38, and 48 were found to be clinically missing. On panoramic radiography, the absence of 12 was confirmed while 13, 18, 22, 28, 38, and 48 were all impacted. A computed tomography (CT) scan revealed a floating anterior maxillary segment with a total of three round/oval oronasal fistulae (Figures 1 and 2).

The treatment objectives were: (1) disimpaction of the impacted teeth; (2) restoration of normal maxillary arch form before bone grafting and palatal defect repair; (3) alleviating crowding in both arches by extraction of 62, 63, 34, and 44; (4) orthodontic traction and alignment of the impacted 13; (5) alveolar bone grafting for closure of the alveolar clefts with consequent closure of the palatal defect using the tongue flap; (6) create adequate space for prosthetic rehabilitation of 21; and (7) long‐term stability.

**FIGURE 3:**
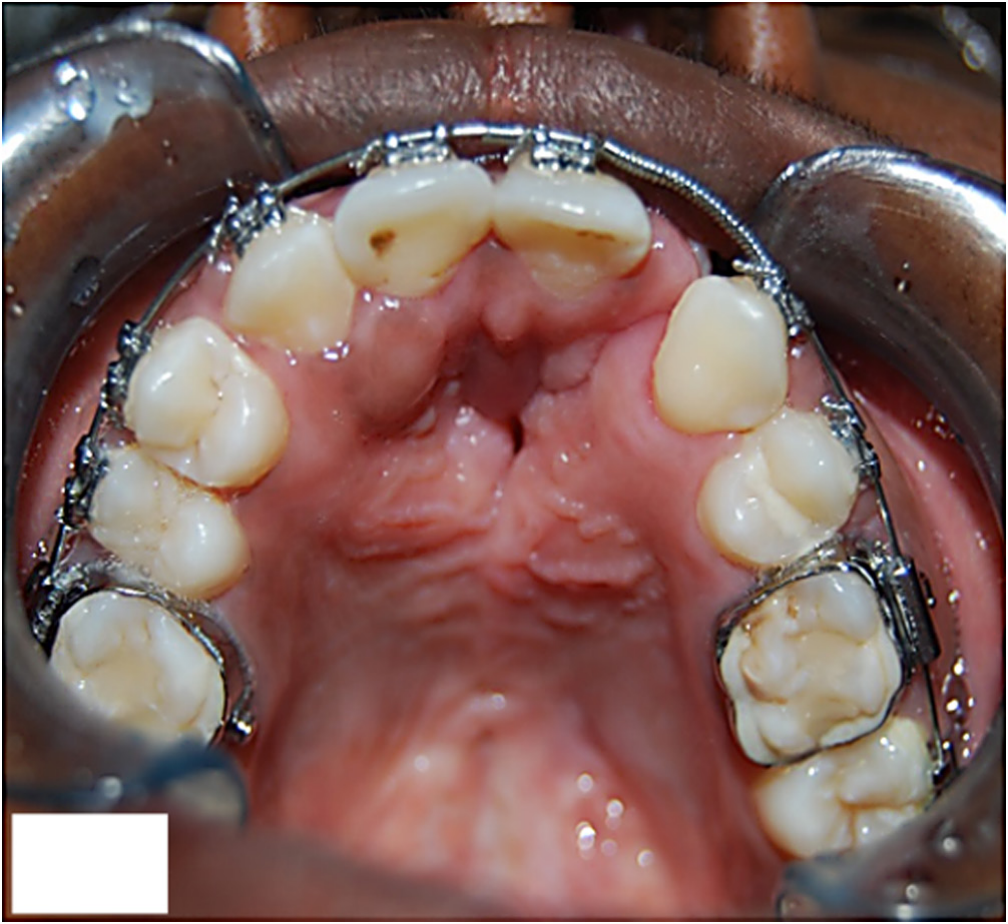
The appearance of the patient after maxillary arch expansion and fixed orthodontic treatment. Note the size of the oronasal communication and cleft extending between the 21 and 23.

The management of this patient was divided into three well‐defined phases: The first phase involved presurgical orthodontics which entailed extraction of 62, 63, and first premolars (34, 44) and was followed by maxillary arch expansion using a hyrax maxillary expander to create enough space to allow realignment of the impacted right maxillary canine (13) using orthodontic traction. Fixed orthodontic appliances were then bonded. After presurgical orthodontics, the final size of the ONC was 3.0 cm at its widest point (Figure 3). This case report focuses on the surgical closure of the ONC. Further details regarding the orthodontic phase of treatment of this case have been published previously by Guthua et al.^8^

**FIGURE 4:**
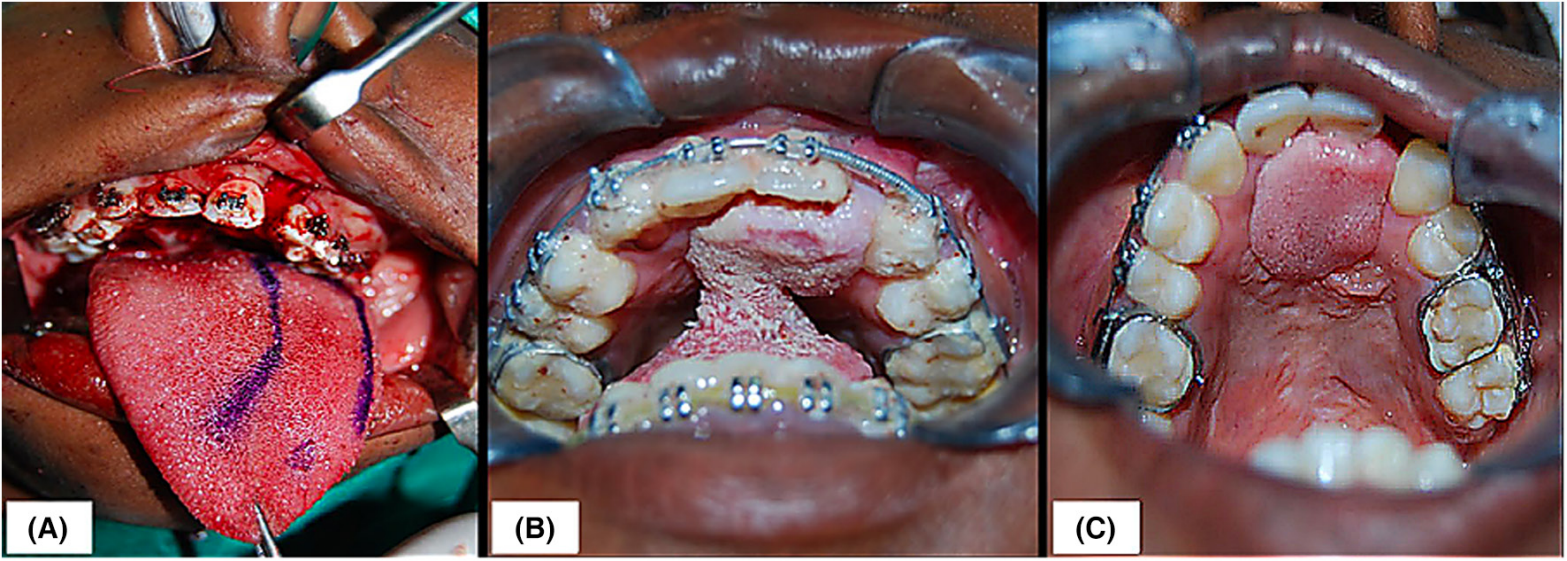
(A) Design of the anteriorly based tongue flap on the left side of the tongue using indelible ink. (B) Intraoral appearance of the tongue flap after suturing of the flap to the margins of the oronasal communication on the anterior palate. (C) Final appearance of the closed ONC after the division of the pedicle of the tongue flap.

The second phase of treatment was directed toward the closure of the alveolar clefts and the palatal ONC. All procedures were performed under general anesthesia with nasotracheal intubation. The wisdom teeth were first extracted, and bone from these sites was harvested in particulate form and used to pack and close the alveolar clefts bilaterally. Incision lines of the fistula were then injected with 2% lidocaine with 1:100,000 adrenaline for hemostasis and ballooning of tissues for ease of dissection. Incisions were made on the margins of the defect and the flaps were meticulously developed and mobilized to avoid tearing. These were sutured together using 4‐0 Vicryl sutures thus constructing the first layer (nasal layer). The anteriorly based tongue flap was delineated and designed on the left side of the tongue such that its base was positioned slightly posterior to the defect when the mouth was closed. This ensured that the pedicle was of adequate length to avoid tension when sutured to the palate. The length and width of the flap were carefully determined so that it was positioned just anterior to the circumvallate papillae and was slightly wider than the width of the defect. Before the elevation of the flap, local anesthetic was infiltrated through the incision lines to control bleeding. The depth of the flap was 5 mm which included a thin layer of muscle which was important as this layer protected the submucosal plexus of the flap. Donor site closure was performed using Vicryl 3‐0 sutures and the flap was rotated upward, advanced to the defect, and a proper edge‐to‐edge approximation of the tongue flap margins to the mucoperiosteal margins was done (Figure 4).

The patient was fed on a nasogastric tube for a period of 3 days after which blenderized feeding was resumed for 1 week and later a soft diet was commenced. After 3 weeks, the pedicle was divided and the posterior aspect of the flap was sutured to the posterior margin of the defect. The excess pedicle was re‐inserted and sutured to the donor site. Postoperative pain was managed using a combination of paracetamol and diclofenac. The antibiotic cover consisted of intravenous augmentin 1.2 g, thrice a day for 72 h and then 1 g peroral twice a day for 2 days. Clinical evaluation after discharge was undertaken at durations of 2 weeks, 1, 3, and 6 months, respectively. The final phase of treatment involved postsurgical orthodontics to close all spaces and coordinate the occlusion. No further complications were reported on follow‐up. Written and verbal consent was provided by the patient for publication purposes.

### 2.2 CASE 2

**FIGURE 5:**
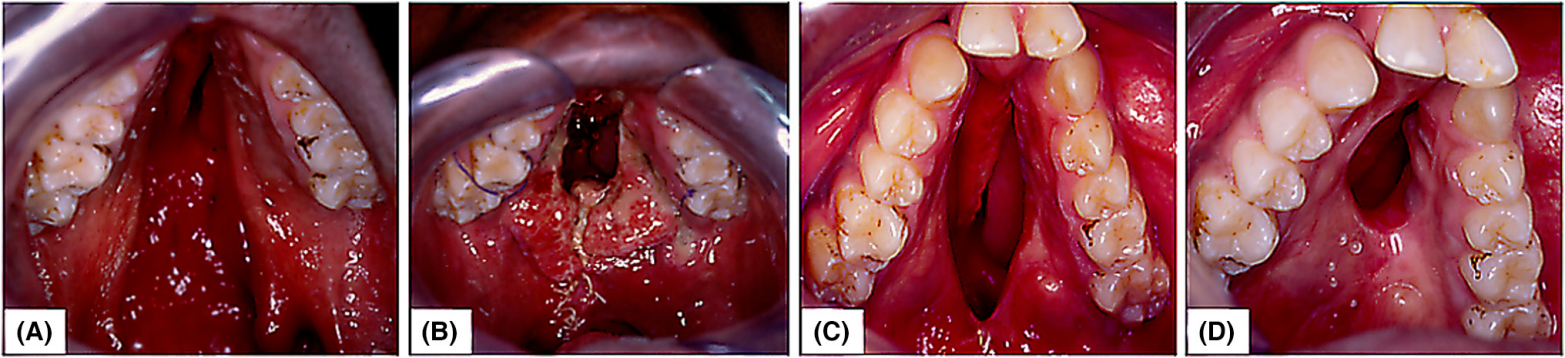
Initial presentation of the oronasal communication secondary to the bilateral cleft palate before any surgical intervention (A), and consequent failure after the first reconstruction effort (B, C). A second surgery reconstructive surgery using local, traditional flaps was attempted which also failed to lead to the presentation shown above (D).

An 18‐year‐old African male was referred to the University of Nairobi Dental Teaching Hospital for treatment for a large, persistent ONC secondary to bilateral cleft lip and palate. The patient's chief complaint was ONC persistence after several surgeries and was dissatisfied with his smile. The patient's medical history included a bilateral cleft lip repaired at 2 months of age and three separate failed attempts at the closure of the palatal defect between 10 and 15 years of age (Figure 5). The patient was healthy and presented with no other comorbidities. Extra‐orally, the patient presented with a convex facial profile, with a symmetrical face accompanied by bilateral scars on the lip consistent with cleft lip repair. Functional examination revealed that the temporomandibular joint was asymptomatic with no shifting of the mandible during opening and closing or deglutition. Intraoral examination revealed a large palatal ONC measuring 4.0 cm along its widest points. Furthermore, bilateral alveolar clefts were also noted to be present between teeth number 11, 13 and 21, 23, respectively. Notably, 12 and 22 were missing. In terms of dental relations, the patient presented a bilateral class I Angles molar relationship, and bilateral class I canine relationship.

**FIGURE 6:**
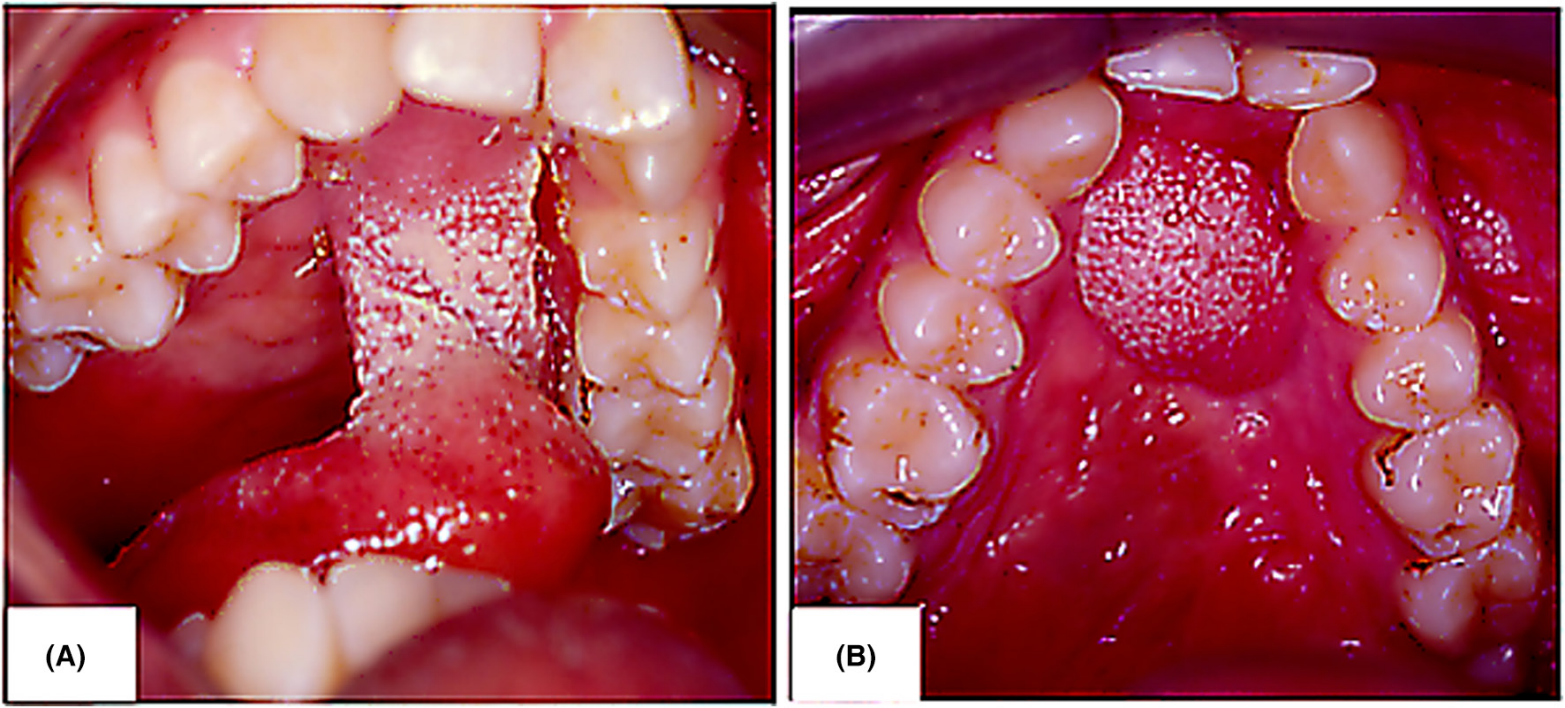
Elevation of anteriorly based tongue flap and suturing to the margins of the defect (A). The final presentation of the closed oronasal communication using the tongue flap (B).

After reviewing all the treatment alternatives, considering the patient's history of numerous surgical failures and the dimensions of the ONC, the use of an anteriorly based dorsal tongue flap was chosen as the preferred option considering its merits in the successful closure of persistent palatal defects. The surgery was performed under general anesthesia when the patient was 18 years old. Similar to the procedure in Case 1, the edges of the defect were injected with lidocaine with adrenaline 1:100,000, and tissues were mobilized and sutured in the midline to achieve closure of the nasal layer. This was followed by designing the flap on the dorsum of the tongue which was elevated and sutured to the recipient site. After a period of 5 days, the patient was discharged. A second surgery was then undertaken after 3 weeks to divide the pedicle and reposition the excess tissue into the donor site. Upon follow‐up, healing at both surgical sites was excellent with no signs of necrosis or relapse of the ONC. The patient reported no further complications (Figure 6). Written and verbal consent was provided by the patient for publication purposes.

## 3 DISCUSSION

The tongue flap was initially described by Eisenberg as a surgical alternative for the repair of intraoral defects. In 1909, Lexer used a modified version of the lateral tongue flap to reconstruct defects of the retromolar trigone and tonsillar fossa. The use of the tongue flap gained widespread attention after Guerrero‐Santos and Altamirano popularized it as a method of palatal defect closure.^9^ Its use was still quite limited as there was reluctance among surgeons for fear it may considerably affect the function of the tongue leading to interferences in speech, taste, and deglutition. Despite the initial reservations of many clinicians, the flap proved to be a safe and effective method for the closure of palatal defects with no such sequelae.^7^ To date, the tongue flap is still not considered a first‐line surgical option for palatal defects; it is, however, indispensable among patients presenting with large, persistent ONCs where other conventional approaches such as local mucoperiosteal flaps have repeatedly failed.^10^

ONCs have an incidence between 4% and 35% and a recurrence rate of 33%–37% after primary surgical closure.^11^ Recurrence rates are found to be much greater among patients with large ONCs that are repaired using local mucoperiosteal flaps alone. The causes of such recurrence may be attributed to suturing flaps under tension, avascular necrosis of the flaps, postoperative infection, and delayed healing.^4^ The management of recurrent or persistent ONCs remains a daunting challenge for the surgeon due to the poor quality of surrounding tissues presenting as a triad of tissue scarring (fibrosis), ischemia, and mucosal irregularity.^7^ Therefore, further attempts at closure by transposition of local flaps may yield extremely low success rates and inevitably lead to repeated failure. The anteriorly based dorsal tongue flap is a safe and effective method for the closure of such defects and it is in these cases that the merits of the tongue flap are fully realized over other types of surgical approaches.^12^

Distant flaps such as tubed pedicle flaps from the abdomen, arm, neck, or cervicothoracic region are a popular option for the closure of palatal defects. Not only is the transfer of oral tissue to the palate rather than skin from distant sites psychologically more acceptable to the patient, but it is also much less cumbersome and less time‐consuming.^7^ In developing countries, every patient cannot be offered free flaps due to the increased cost, increased operating time, and lack of expertise.^13^ Furthermore, such flaps result in a considerable amount of donor site morbidity which predisposes the patient to more complications as opposed to having a single surgical site located within the oral cavity.

The tongue flap offers several advantages such as localization of the surgical site to the oral cavity, location of the donor site and recipient site in close proximity to one another, abundant tissue with an excellent blood supply, ease of rotation, and versatility in flap design and elevation.^12^ Furthermore, there is a significant reduction in operating time as the flap is easy and quick to harvest, taking less than 30 min in our cases. This in turn translates to less cost for the patient as opposed to the distant flaps. Unlike the lateral tongue flap, the floor of the mouth is not included in the dorsal tongue flap hence tethering or fixation of the tongue does not occur, and therefore speech is not affected.^13^ Furthermore, the circumvallate papillae are not crossed, so swallowing remains intact. With the use of this technique, it is important to keep in mind that an adequate pedicle length allows greater movement of the tongue hence increasing patient comfort and a minimum flap thickness of 5 mm should be used to allow the incorporation of the submucosal plexus in the flap. This ensures reliable vascularity and healing. In addition to the management of congenital defects of the palate as described above, the tongue flap may even be employed in traumatic impairments of the palate (acquired defects),^14^ thus potentially making it an alternative first‐line consideration in the management of palatal defects. This hypothesis is supported by the fact that the flap is associated with a relative lack of complications and has shown high success rates among our cases as well as those reported by others hence preserving the limited resources of these patients. Success rates varying from 85% to 95.5% have been reported provided that the case selection is genuine.^2^, ^15^, ^16^ As the flap is interpolated and must be maintained this way for 2–3 weeks, it may not be ideal in young children or among children with special needs as there may be uncontrolled tension on the flap which may lead to detachment from the palate.^17^

Some of the drawbacks of the tongue flap are the difficulties in intubation and extubation intraoperatively, limited intraoral function, and risk of detachment.^12^, ^18^ Additionally, it is a two‐staged procedure that requires the division of the pedicle after a period of 2–3 weeks. This may result in some clinicians advocating for the use of distant flaps instead.^17^ Our view is that patient discomfort due to the interpolation between the donor and recipient sites may be a small price to pay compared to the tongue flap's great versatility and its success in the closure of persistent ONCs where alternative methods of closure have failed.

## 4 CONCLUSION

Large, persistent ONCs as a result of previously failed surgical repair are extremely challenging to manage successfully due to the poor local soft tissue profile surrounding the defect. Repair using local mucoperiosteal flaps alone may lead to recurrent failure and therefore an alternative technique is mandatory. The anteriorly based dorsal tongue flap is a simple, reliable flap for the successful closure of such defects, yielding satisfactory outcomes with high success rates. Due to certain limitations of the tongue flap, good case selection is key. Nonetheless, future large‐scale studies are needed for a definitive recommendation and conclusion.

## AUTHOR CONTRIBUTIONS

**Symon Guthua:** Conceptualization; investigation; methodology; supervision; writing – review and editing. **Krishan Sarna:** Data curation; formal analysis; methodology; writing – original draft; writing – review and editing. **Martin Kamau:** Methodology; supervision; writing – review and editing. **Peter Ng'ang'a:** Investigation; methodology; supervision; writing – review and editing.

## FUNDING STATEMENT

There was no source of funding for this study.

## CONFLICT OF INTEREST

The authors declare that there is no conflict of interest.

## PROFESSIONAL MEETING PRESENTATION

The work contained in this manuscript was presented at the 39th Kenya Dental Association Scientific Conference and Exhibition. Leveraging technology in oral healthcare Naivasha, Kenya, October 27–29, 2022.

## ETHICS STATEMENT

The manuscript was prepared according to standard publication ethical guidelines.

## CONSENT

The authors confirm that signed consent was obtained from the patient prior to publication.

## Data Availability

The data that support the findings of this study are available on request from the corresponding author. The data are not publicly available due to privacy or ethical restrictions.
